# Transcriptomic Analysis of Polyhexamethyleneguanidine-Induced Lung Injury in Mice after a Long-Term Recovery

**DOI:** 10.3390/toxics9100253

**Published:** 2021-10-08

**Authors:** Jeongah Song, Kyung-Jin Jung, Jae-Woo Cho, Tamina Park, Su-Cheol Han, Daeui Park

**Affiliations:** 1 Animal Model Research Group, Korea Institute of Toxicology, Jeongeup 56212, Korea; 2 Bioanalytical and Immunoanalytical Research Group, Korea Institute of Toxicology, Daejeon 34114, Korea; jungk@kitox.re.kr; 3 Toxicologic Pathology Research Group, Korea Institute of Toxicology, Daejeon 34114, Korea; cjwoo@kitox.re.kr; 4 Department of Predictive Toxicology, Korea Institute of Toxicology, Daejeon 34114, Korea; tamina.park@kitox.re.kr; 5 Department of Human and Environmental Toxicology, University of Science and Technology, Daejeon 34113, Korea; 6 Jeonbuk Department of Inhalation Research, Korea Institute of Toxicology, Jeongeup 56212, Korea; vethansc@kitox.re.kr

**Keywords:** polyhexamethyleneguanidine, persistent inflammation, lung dysfunction, transcriptome

## Abstract

Polyhexamethyleneguanidine phosphate (PHMG-P) is one of the causative agents of humidifier disinfectant-induced lung injury. Direct exposure of the lungs to PHMG-P causes interstitial pneumonia with fibrosis. Epidemiological studies showed that patients with humidifier disinfectant-associated lung injuries have suffered from restrictive lung function five years after the onset of the lung injuries. We investigated whether lung damage was sustained after repeated exposure to PHMG-P followed by a long-term recovery and evaluated the adverse effects of PHMG-P on mice lungs. Mice were intranasally instilled with 0.3 mg/kg PHMG-P six times at two weeks intervals, followed by a recovery period of 292 days. Histopathological examination of the lungs showed the infiltration of inflammatory cells, the accumulation of extracellular matrix in the lung parenchyma, proteinaceous substances in the alveoli and bronchiolar–alveolar hyperplasia. From RNA-seq, the gene expression levels associated with the inflammatory response, leukocyte chemotaxis and fibrosis were significantly upregulated, whereas genes associated with epithelial/endothelial cells development, angiogenesis and smooth muscle contraction were markedly decreased. These results imply that persistent inflammation and fibrotic changes caused by repeated exposure to PHMG-P led to the downregulation of muscle and vascular development and lung dysfunction. Most importantly, this pathological structural remodeling induced by PHMG-P was not reversed even after long-term recovery.

## 1. Introduction

An increase in acute lung injury in pregnant women was reported by the Korea Center for Disease Control and Prevention (KCDC) in 2011. Epidemiological investigation and toxicological research revealed that humidifier disinfectants were the cause of the lung injuries [1]. Humidifier disinfectants are used to prevent the growth of microorganisms in humidifier water tanks. The aerosolized disinfectants enter human lungs and induce inflammatory and fibrotic responses, resulting in progressive interstitial pneumonia with fibrosis [2]. A humidifier disinfectant product containing chloromethylisothiazolinone/methylisothiazolinone was first introduced to the Korean market in 1994. Products containing polyhexamethyleneguanidine phosphate (PHMG-P) and oligo(2-(2-ethoxy)-ethoxyethyl guanidinium chloride were marketed in 2001 and 2009, respectively [3,4]. The use of humidifier disinfectants has increased as public awareness regarding hygiene issues increased over time [5]. Humidifier disinfectant-induced lung injury cases have been reported since 2006 [6] and 4114 people were recognized with humidifier disinfectant-related injuries by December 2020 [7]. Among humidifier disinfectants, PHMG-P had the highest usage rate (62.0%) and the number of related patients was concurrently high [4]. 

Patients with humidifier disinfectant-associated health issues showed bronchiolar destruction with alveolar destruction in the early phase and displaced parenchymal architecture due to inflammation and fibrosis in the late phase [8,9]. Hur et al. reported that centrilobular nodules, fibrosis and bronchiectasis were observed at least five years after the onset of this lung injury [8]. Patients also showed persistent severe restrictive lung function. These adverse outcomes showed a good correlation with the intensity of humidifier disinfectant exposure [2,9,10]. The onsets of other complications such as liver damage or lung cancer in humidifier disinfectant-associated patients 20 or more years after exposure is still unknown. Further studies including epidemiological studies are needed to identify the pathophysiology of humidifier disinfectant-associated lung injuries and the possible health effects of exposure to humidifier disinfectants later in life. 

RNA sequencing (RNA-seq) is a recently developed sequencing technique. Complementary DNAs synthesized from RNA are sequenced and aligned to a reference genome. This technique can precisely quantify the transcript levels of the genes of interest [11]. It can be used to track gene expression changes and understand the transcriptomic dynamics between diseased and normal tissue [12]. Since the transcriptome sensitively responds to toxicants, the analysis of expression profiling of the transcriptome is useful to understand the molecular mechanisms and predict disease progression in organisms after toxicant exposure. In addition, some researchers [11,13] have showed that distinct blood gene expression profiles reflected hepatotoxicity earlier than traditional toxicity endpoints such as histopathological, hematological and serum biochemical changes in acetaminophen-induced rats. Therefore, these profiles can be used as sensitive biomarkers. These biomarker genes were also significantly changed earlier than serum biochemical parameters in humans [13,14,15,16]. Thus, these findings support that transcriptome analysis is a powerful tool for identifying toxicity and predicting the progression of toxicant-induced damage as well as the potential adverse health effects of toxicant exposure.

In this study, we investigated whether lung damage following repeated exposure to PHMG-P was reversed after a long-term recovery and evaluated the adverse effects of PHMG-P on mice lungs when the lung injury did not resolve. We performed histopathological examinations to check the morphological and structural changes of the lungs and conducted RNA-seq to investigate the biological changes at the molecular level. Our findings shed light on the pathogenesis of PHMG-P injury and the molecular mechanism of action underlying PHMG-P-induced lung damage.

## 2. Materials and Methods

### 2.1. Materials

PHMG-P solution (25.1%, CAS No: 89697-78-9) was purchased from BOC Sciences (Shirley, NY, USA). Saline was obtained from Daihan Pharmaceutical Co. (Ansan, Korea).

### 2.2. Animals

Seven-week-old male C57BL/6 mice were purchased from Orient Bio, Inc. (Seongnam, Korea) and maintained in an environmentally controlled animal room (19–25 °C, 40–60% humidity, air ventilation at 10–20 times/h with a 12 h light/dark cycle). The experimental animals were fed with sterile pelleted food (PM Nutrition International, Richmond, VA, USA) and UV-irradiated (Steritron SX-1; Daeyoung, Inc., Seoul, Korea), filtered (1 µm) tap water was provided ad libitum. The mice were acclimatized for seven days. All experiments were approved by the Institutional Animal Care and Use Committee of the Korea Institute of Toxicology and conducted according to the Association for Assessment and Accreditation of Laboratory Animal Care International guidelines.

### 2.3. Experimental Design

The mice were randomly divided into two groups. The control group and the treated group each consisted of 10 mice. The mice received an intranasal instillation of PHMG-P (0.3 mg/kg, 50 µL/animal) six times at 2-week intervals. The control group was instilled with saline in the same way. Two hundred and ninety-two days after the last instillation (363 days after the first instillation), all mice were euthanized with an overdose of isoflurane. The experimental scheme is shown in Supplementary Figure S1. The lungs were removed and weighed. The left lung was fixed in 10% neutral buffered formalin and the right lungs were stored in RNAprotect Tissue reagent (Qiagen, Hilden, Germany) for further RNA extraction. 

The dose of PHMG-P was determined based on a previous epidemiological study [2]. According to the study conducted by Paek et al. (2015) [2], the average total duration of humidifier disinfectant exposure in definite cases was 18.4 months and the duration of use per day was 11 h. The concentration of PHMG-P aerosol particle exposure was approximately 0.1 mg/m^3^ (KCDC 2011). The total uptake dose by humans was calculated from the equation recommended by the Association of Inhalation Toxicologists [17]. The equation is as follows:

Delivered dose (mg/kg) = $\frac{\mathrm{Concentration}\;\mathrm{of}\;\mathrm{substance}\;\mathrm{in}\;\mathrm{air}\;\left(\frac{\mathrm{mg}}{L}\right)\times \;\mathrm{Respiratory}\;\mathrm{Minute}\;\mathrm{Volume}\;\times \;\mathrm{Duration}\;\mathrm{of}\;\mathrm{exposure}\;\left(\min\right)}{\mathrm{Body}\;\mathrm{weight}\;\left(\mathrm{Kg}\right)}$. The calculated uptake dose by humans was 3.6 mg/kg and was translated to a dose for mice (43.2 mg/kg) based on body surface area [18]. The mice were administered a total dose of 1.8 mg/kg PHMG-P, which was 24 times lower than the estimated human exposure dose. The single instillation dose of PHMG-P (0.3 mg/kg) was determined from our previous experiment [19]. 

### 2.4. RNA Extraction and mRNA Analysis

To validate the RNA-seq data, real-time polymerase chain reaction (PCR) was performed. Total RNA was extracted using the RNeasy Mini Kit (Qiagen, Hilden, Germany). The RNA concentration and purity were measured using a Nanodrop ND-1000 spectrophotometer (Nanodrop Technologies, Wilmington, DE, USA). RNA samples with A260/280 and A260/230 ratios of >1.8 were used. One microgram of total RNA was reverse transcribed to cDNA using the GoScript™ Reverse Transcription System (Promega, Madison, WI, USA). Arg1, Arg2, Cav1, Ctsb, Gucy1a1, Ccl2, Mmp12, Ptges, Rab7b, Krt14 and Serpine1 mRNA levels were measured using the ABI QuantiStudio 5 (Applied Biosystems, Woolston, Warrington, UK). The sequences of the primers are shown in Supplementary Table S1. The specificity of the primers was validated by checking the efficiency of the standard curve, the melting curve and agarose gel electrophoresis. PCR reactions were performed using SYBR Green PCR Master Mix (Applied Biosystems, Woolston, Warrington, UK). The PCR conditions used were as follows: 95 °C for 10 min, followed by 40 cycles of 15 s at 95 °C and 1 min at 60 °C. The relative expression levels were calculated using the comparative threshold cycle (delta–delta CT) method compared to the control and were presented as fold changes [20]. Hprt was used as the internal control. All reactions were carried out in three or four biological replicates and two technical replicates. 

### 2.5. Histopathological Examination

The lungs were fixed in 10% neutral formalin buffer for histological examination. The fixed samples were cut into 4-µm-thick paraffin sections and subjected to hematoxylin and eosin (H&E) staining and Masson’s trichrome staining for microscopic observation. Two or three tissue sections per mouse were examined by microscopic observation. The degree of inflammation or fibrosis was evaluated on a subjective scale of 0–5, where 0 indicated the absence of inflammation and fibrosis, 1 the presence of inflammation and fibrosis involving < 20% of the lung parenchyma, 2 the lesions involving 20–40% of the lung, 3 lesions involving 40–60% of the lung, 4 lesions involving 60–80% of the lung and 5 lesions involving >80% of the lung.

### 2.6. RNA Extraction and Preparation for Illumina Nova-Seq Sequencing

The lungs of three mice per group were subjected to RNA-seq. Total RNA was isolated from the lung tissue prepared as described above. An RNA-seq library was generated using the TruSeq Stranded Total RNA LT Sample Prep Kit according to the manufacturer’s instruction (Illumina, San Diego, CA, USA). Briefly, mRNA was separated from total RNA using oligo(dT) beads and chemically fragmented. After double-strand cDNA synthesis of the fragmented mRNA, end-repair, adenylation of the 3′-end and sequencing adapter ligation were performed, followed by DNA purification with magnetic beads and PCR amplification. Finally, the amplified library was purified, quantified and applied for template preparation. The NovaSeq 6000 platform was utilized to generate 101-bp paired-end sequencing reads (Illumina).

### 2.7. Genome Mapping of Paired-End Sequences and Differentially Expressed Genes

All 101-bp paired-end sequence reads were mapped to the reference genome sequences for *Mus musculus* (UCSC mm10) using HISAT2 version 2.1.0 [21]. The mapped reads were assembled using StringTie version 1.3.4 [22] and the reads were merged for each condition (control group and PHMG-P-treated group). The reads for each condition were quantified using the read count and fragment per kilobase of transcript per million mapped reads (FPKM). Finally, we identified the differentially expressed genes (DEGs) with the two-fold changes and independent *t*-test *p*-values under 0.05. In addition, the expression volume indicated the expression intensity, which was defined as the geometric mean of the expression values between the control group and PHMG-P-treated group.

### 2.8. Gene Set Enrichment Test

To characterize the biological pathways related to DEGs, the representative pathways were analyzed in the context of the Metascape web portal [23]. Metascape analyzes the inference of enriched biological pathways and the enrichment clustering of biological pathways based on various resources curated from independent knowledgebases including gene ontology [24]. Metascape automatically clusters the enriched biological pathways into non-redundant groups, where it implements a similar function. For example, pairwise similarities between any two enriched biological pathways are computed based on a Kappa-test score [25]. The most significant pathways with a *p*-value of less than 0.01 were clustered into each group based on pairwise similarities. The hypergeometric test and the Benjamini-Hochberg *p*-value correction algorithm were used to identify all biological pathway terms that contained a statistically greater number of genes in common with an input list than was expected by chance.

### 2.9. Sample Clustering and Heatmap 

To check the relationship between the control group and PHMG-P-treated group, we calculated Euclidean distances and complete linkage as the agglomeration method for hierarchical clustering based on DEGs. The heatmap images were generated in R with the heatmap.2 function in the gplots library (https://cran.r-project.org/web/packages/gplots/, access date: 2 June 2021)

### 2.10. Data Analysis

The Student’s *t*-test was used to determine the differences in the mRNA expression levels in lung tissue from the control and PHMG-P-treated groups (SPSS Ver15.0.0, SPSS Inc., Chicago, IL, USA). A *p*-value less than 0.05 was considered as statistically significant. All data are presented as mean ± standard deviation. The number of samples in each group is indicated in the figure legends. In the analysis of the RNA-seq data, the statistical method was described in each result. 

## 3. Results

### 3.1. Histopathological Examination

**Figure 1:**
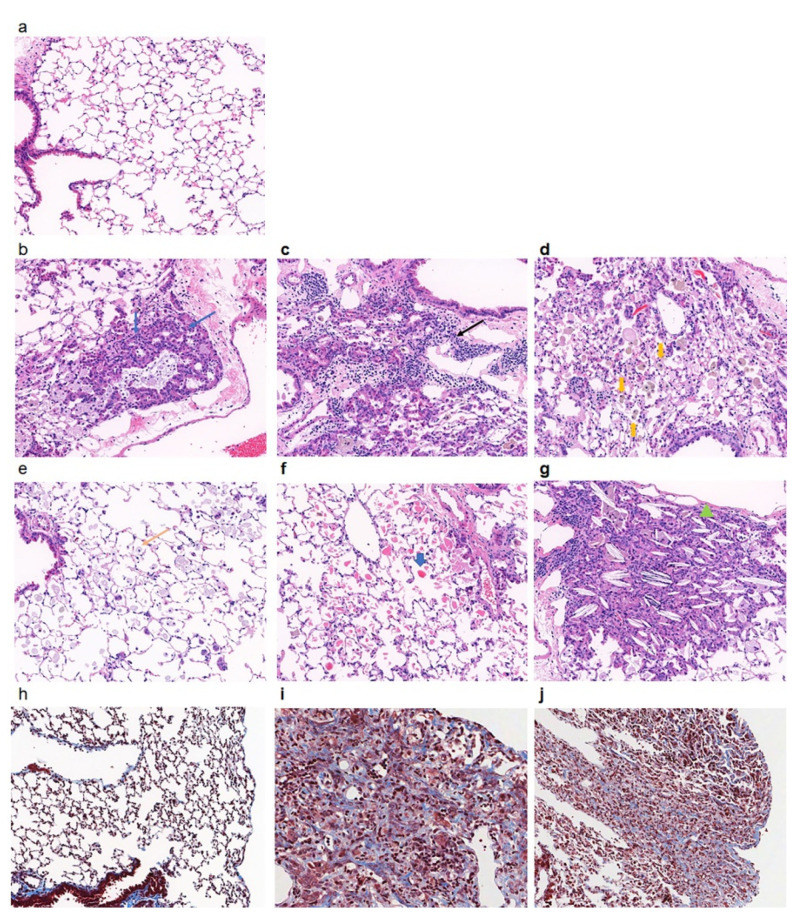
Histopathological analysis of lungs of mice instilled with PHMG-P. Representative histological sections were shown from control (**a**,**h**) and PHMG-P-treated (**b**–**g**,**i**,**j**) groups. The lung sections were stained with hematoxylin and eosin (**a**–**h**) and Masson’s Trichrome (**h**–**j**). Blue arrow indicates hyperplasia of epithelial cells (**b**), black arrow indicates lymphocyte infiltration (**c**), yellow thickened arrow indicates pigmented macrophage (**d**), yellow arrow indicates foamy macrophage (**e**), blue thickened arrow indicates proteinaceous substance (**f**) and filled green triangle indicates crystal cleft (**g**) (original magnification, ×200).

The morphological changes in the lungs are presented in Figure 1. In the PHMG-P-treated group, the infiltration of inflammatory cells, interstitial fibrosis and bronchioloalveolar hyperplasia were observed (Figure 1 and Table 1). Lymphocytes and alveolar macrophages accumulated multifocally in the perivascular connective tissue and peribronchiolar and interstitial areas of the lungs. Hyperplasia of type II epithelial cells and bronchiolization were accompanied with foamy macrophages. In addition, pigmented macrophages and an eosinophilic proteinaceous substance were commonly seen in the alveolar space. Cholesterol cleft with neutrophilic infiltration was seen in the alveoli of 75% of mice from the PHMG-P-treated group. The source of the proteinaceous substance and cholesterol cleft are known to be pulmonary surfactants produced by type II alveolar cells or lipids released from destroyed alveolar cells [26,27,28]. In Masson’s trichrome staining, collagen deposition was seen and predominant in the lung parenchyma of mice from the PHMG-P-treated group (Figure 1h–j).

From the present study, findings related to edema, hemorrhage, neutrophilic infiltration, hyaline substance in the cytoplasm of hyperplastic alveolar epithelial cells, mucous excretion and cellular debris in the bronchial lumen, mineralization in the interstitium and the protrusion of fibroblastic foci into the bronchiolar space were relatively unusual and might be associated with subsequent response to inflammation. 

### 3.2. Gene Expression Profiles in PHMG-P-Treated and Control Groups

**Figure 2:**
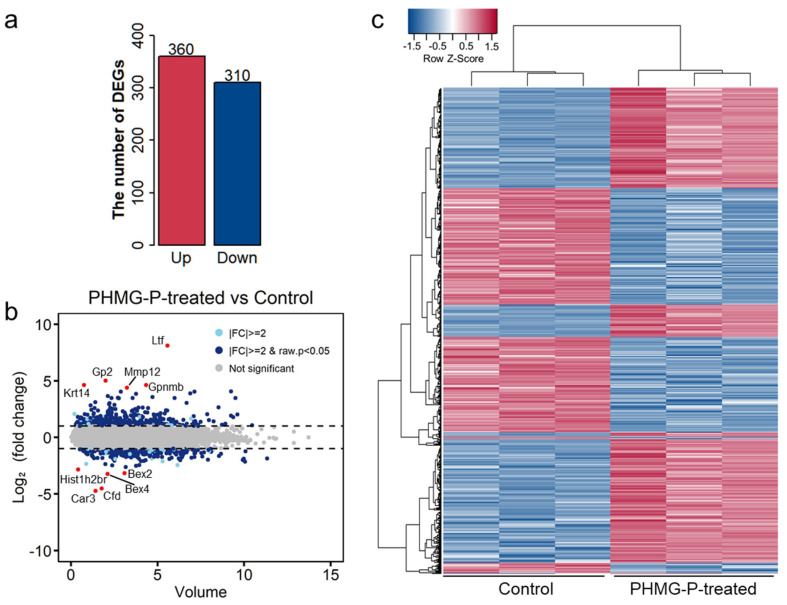
Distribution of differentially expressed genes by PHMG-P treatment. (**a**) The number of DEGs in the PHMG-P-treated group compared to the control group. (**b**) An overview of the DEGs is depicted by MA plot. (**c**) Heatmap based on the Euclidean distances of the DEGs.

To better understand the mechanism of the adverse effects of PHMG-P, we implemented genome-scale analysis of the gene expression profiles. First, we analyzed the DEGs in the PHMG-P-treated and control groups using RNA-seq based on the next-generation sequencing method. RNA-seq analysis revealed 670 genes differentially expressed between the control and PHMG-P-treated groups (fold change > 2 and *p* < 0.05). Of the 670 DEGs, 360 genes were significantly upregulated and 310 were significantly downregulated (Figure 2 and Supplementary Table S2).

The highly upregulated genes in the PHMG-P-treated group were *Ltf, Krt14, Gp2, Gpnmb, MMP 12* and *Saa3* (Table 2 and Figure 2b). Among these, Ltf, MMP12 and Sas3 are associated with immune response or inflammation. Lactotransferrin (Ltf) is a secondary mediator excreted by activated neutrophils [29] and MMP12 is a metalloproteinase produced by macrophages [30]. Saa3 is a biomarker of inflammation or injury and one of the acute phase proteins [31].

The highly downregulated genes were *Bex4*, *Bex2*, *Fabp1* and *Hist1h2br* (Table 3 and Figure 2b). Based on clustering analysis, the control and PHMG-P-treated groups were well divided (Figure 2c). In addition, the heatmap of the DEGs showed significant differences between the control and PHMG-P-treated groups. (Figure 2c).

### 3.3. Gene Set Enrichment Analysis of PHMG-P Treatment

**Figure 3:**
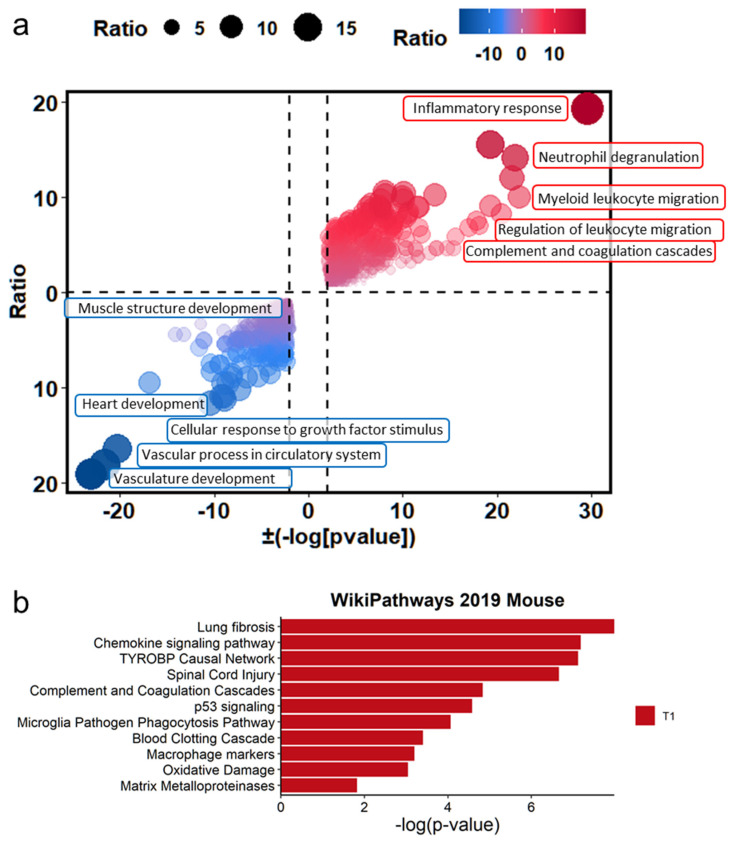
The difference in biological signatures between the control and PHMG-P-treated groups. (**a**) Gene set enrichment test based on gene ontology categories. (**b**) Biological pathways significantly changed by PHMG-P treatment. The significant *p*-values were converted to −log(*p*-value).

To identify the significant biological pathways associated with PHMG-P treatment, gene set enrichment analysis of the DEGs was conducted. The biological pathways were analyzed using Metascape (https://metascape.org, access date: 10 May 2021)) [23] and EnrichR [32]. In Metascape, DEGs were subjected to gene ontology analysis to identify the different changes resulting from PHMG-P treatment. The statistical significance (*p*-value) of each pathway was calculated using Fisher’s exact test. The plots of pathways filtered according to a *p*-value of less than 0.01 are shown in Figure 3. The significant pathways based on the Metascape results were plotted with the ratio of the number of mapped genes to the total number of related genes in the pathways (Figure 3a). In addition, the ten most significant pathways were selected in the order of *p*-value (Supplementary Figure S2). Immune response-related pathways such as inflammatory response (29.50 −log2 (*p*-value)), myeloid leukocyte migration (22.38 −log2 (*p*-value)), neutrophil degranulation (21.93 −log2 (*p*-value)), regulation of leukocyte migration (11.74 −log2 (*p*-value)) and complement and coagulation cascades (10.89 −log2 (*p*-value)) were upregulated by PHMG-P treatment (Supplemental Table S3). In addition, EnrichR results showed that PHMG-P treatment was associated with lung fibrosis, chemokine signaling, complement and coagulation cascades, macrophage markers and matrix metalloproteinase in the lung tissue (Figure 3b and Supplementary Table S4). 

In addition, muscle and vascular tissue-related pathways including vasculature development (−23.08 log2 (*p*-value)), vascular process in circulatory system (−11.12 log2 (*p*-value)), heart development (−10.45 log2 (*p*-value)), cellular response to growth factor stimulus (−9.26 log2 (*p*-value)) and muscle structure development (−8.96 log2 (*p*-value)) were associated with DEGs downregulated by PHMG-P (Supplementary Table S3). Usually, pulmonary fibrosis caused by environmental particles and chemicals progresses through the injury, inflammation and repair stages including wound contraction and tissue regeneration. However, the PHMG-P-treated group showed interrupted muscle and vascular system development, which was closely related to tissue regeneration and repair. 

### 3.4. Network Analysis of Gene Sets in PHMG-P Treated Mice

**Figure 4:**
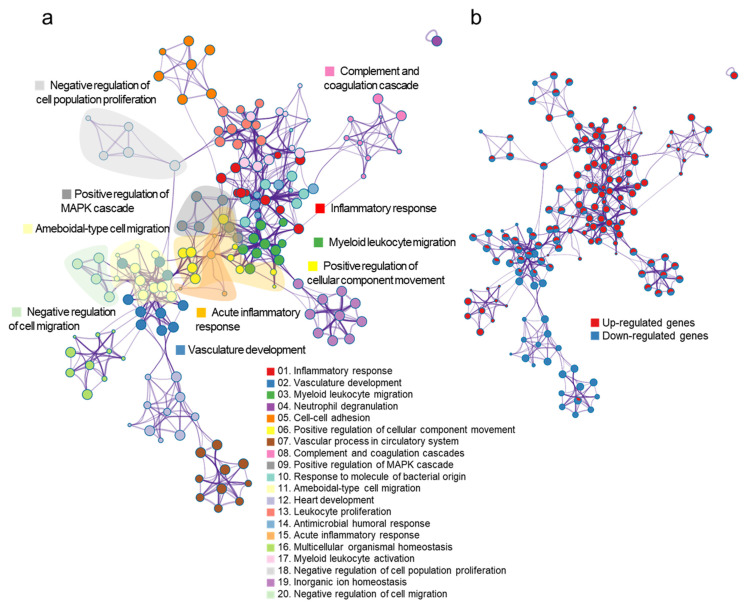
The network of gene sets in the DEGs following PHMG-P treatment. The node in the network indicates a gene set and the edge represents the interaction between gene sets. (**a**) Functional categories of gene sets in the network. The color of the node indicates the functional category of the gene set. (**b**) Expression pattern of gene sets in the network. The node color represents the expression information of the gene set. Red colors indicate the upregulated gene sets and blue colors indicate the downregulated gene sets.

Based on the RNA-seq results, PHMG-P affected inflammation and the dysfunction of muscle and vascular systems. To find the interconnected pathways bridging inflammation and the dysfunction of muscle and vascular systems, we conducted a network analysis of the gene sets in DEGs in PHMG-P-treated mice using the Metascape program. In Figure 4a, the major interconnected pathways between the upregulation of inflammation and the downregulation of muscle and vascular systems were cellular component movement and cell population proliferation (Supplementary Table S5). The cellular component movement were directly connected with inflammation and muscle and vascular system. However, cell population proliferation was indirectly interacted with two pathways through leukocyte proliferation. Especially, the negative regulation of cell population proliferation-related genes was involved in TGF-β regulation of the ECM including *Cdkn2a*, *Cav1*, *Igf1*, *Tgfbr*, *Cdh*, *Lgals3*, *Il6*, *Gpnmb*, *Dlc1*, *Cyp1b1* and *Pde5a* (Figure 4b, Supplementary Tables S2 and S5). In the positive regulation of cellular component movement pathway, *Fga*, *F3*, *F7*, *Vtn*, *Serpine* and *Itgax* genes were closely related to the complement and coagulation cascades. 

### 3.5. Quantitative Real-Time PCR

**Figure 5:**
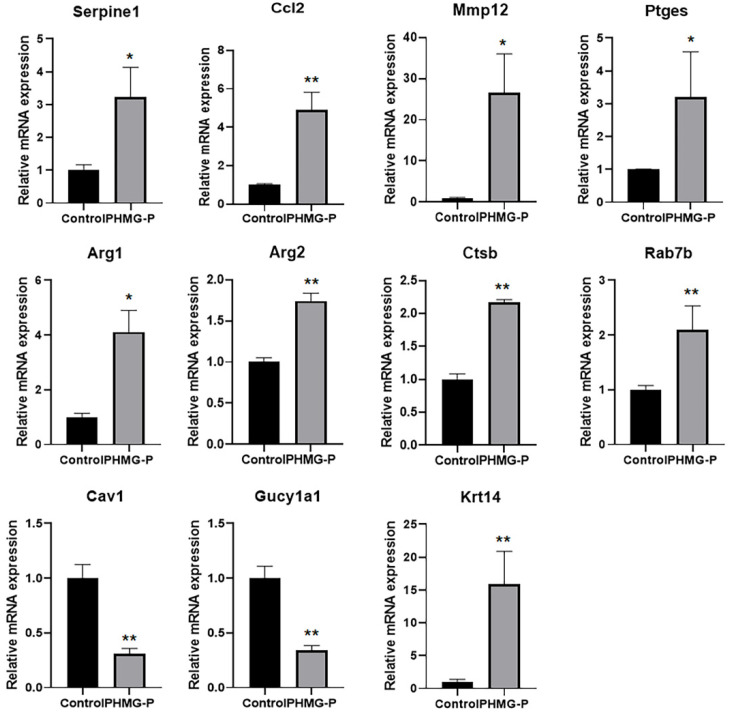
Changes in mRNA expression in the lung tissue of mice following instillation of PHMG-P. Each mRNA was normalized to Hprt and calculated as fold-induction relative to control. The results are expressed as the mean ± SD (*n* = 3–4). The values were significantly different from the control group, * *p* < 0.05 and ** *p* < 0.01.

Real-time PCR was conducted to validate the transcript levels in the RNA-seq data (Figure 5). Consistent with the RNA-seq data, the expression of genes associated with the inflammatory response (*Mmp12*, *Serpine1*, *Ccl2*, *Ptges*), phagosomes and lysosomes (*Ctsb*, *Rab7b*) and fibrosis (*Arg1*, *Arg2*) was significantly increased, whereas the induction of genes associated with smooth muscle development (*Cav1*, *Gucy1al*) and angiogenesis (*Cav1*) was markedly reduced. In addition, *Krt14* gene, one of the top ten upregulated genes, was highly expressed in both groups. 

### 3.6. Macrophage Polarization in the PHMG-P-Treated Group

**Figure 6:**
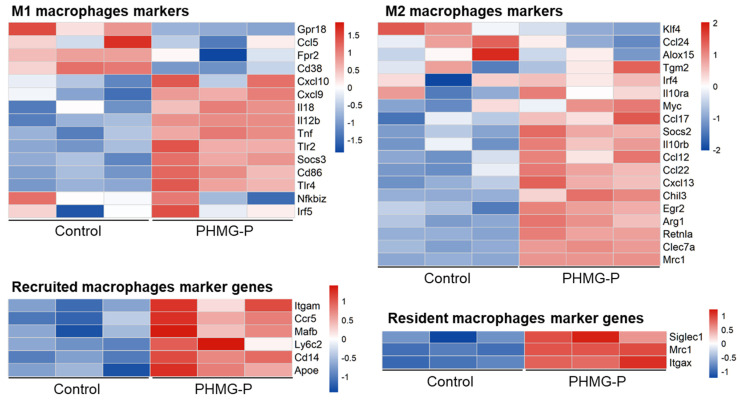
The expression pattern of macrophage polarization and residence-related markers in the control and the PHMG-P-treated groups.

Macrophages play multiple roles in the progression of diseases. To determine the function of alveolar macrophages in the lungs of the control and PHMG-P-treated group, we analyzed the expression of the marker genes of the resident or recruited alveolar macrophages and classical (M1) or alternatively activated (M2) macrophages. Inflammatory responses vary in different fibrotic conditions but share polarization toward a M2 macrophage-mediated response, with the abundant release of profibrotic mediators as a common feature [33]. To evaluate macrophage polarization in the lung tissue of mice from the PHMG-P-treated group, we compared the gene expression patterns of M1 and M2 polarization markers in the control and PHMG-P-treated groups (Figure 6). The marker genes of each subset were chosen from the reports of Mould et al. and Gundra et al. [34,35,36]. 

M2 markers were upregulated by PHMG-P treatment (*p* < 0.01, the Student’s *t*-test). The M2 makers were *Ccl22*, *Ccl12*, *Ccl17*, *Arg1*, *Clec7a* and *Egr2* (Supplementary Table S6). The results indicated that PHMG-P promoted fibrosis by M2 macrophage-mediated response in lung tissue. However, there was no statistical significance in M1 markers expression. In addition, we also compared the macrophage markers related to recruited alveolar macrophages and resident alveolar macrophages. Usually, most of the macrophages that accumulate at diseased sites are derived from circulating monocytes [37]. Both recruited and resident macrophage markers were upregulated in the PHMG-P-treated group (*p* < 0.01, the Student’s *t*-test). The recruited alveolar macrophages makers were *Cd14, Mafb*, *Ccr5*, *Itgam*, *Ly6c2* and *Apoe*. The resident alveolar macrophages markers were *Siglec1*, *Mrc1* and *Itgax*. 

## 4. Discussion

PHMG, a polymeric guanidine family biocide, is one of the major active ingredients of humidifier disinfectants. Humidifier disinfectants containing PHMG-P are commonly used and humidifier disinfectant-associated lung injury, leading to interstitial pneumonitis with pulmonary fibrosis, was frequently reported in people exposed to PHMG-P [4]. Our previous studies showed that pulmonary fibro-inflammatory injury in mice was sustained for at least 10 weeks after a single instillation of PHMG-P into the lungs. The infiltration of immune cells, accumulation of myofibroblasts in the lungs and increased proinflammatory cytokines with activation of the NALP3 inflammasome were detected. Moreover, when mice were intratracheally instilled with 0.3 mg/kg PHMG-P, proinflammatory cytokines and pathological changes were not observed on day 7 but increases in IL-1β production and infiltration of immune cells were detected on day 14 [19]. These results imply that once PHMG-P enters the lungs, it is not eliminated and irritates lung cells, provoking severe and fatal damage. This assumption was proved by [38], who evaluated the biodistribution of PHMG-P by labeling it with radioactive indium (111In). As a result, approximately 74% of the inhaled or 45% of the intratracheally instilled PHMG-P remained in the lungs for seven days, whereas orally ingested PHMG-P was not detected in the lungs and was rapidly expelled within 18 h [38]. These results imply that humidifier disinfectant-associated lung disease will persist in patients a long time after exposure. To verify this, we investigated whether lung damage following repeated exposure to PHMG-P was reversed after a long-term recovery. Based on studies conducted by Dutta et al. (2016) [39] and Wang et al. (2020) [40], the age of the mouse was converted to the age of a human. PHMG-P was first instilled intranasally on 56-day-old mice, which is equivalent to 15.3-year-old humans. The 6th instillation was performed on 126-day-old mice, which is equivalent to 21.5-years-old humans. Necropsy was performed on 419-day-old mice, which is equivalent to 47.5-years-old humans. In conclusion, lung injury resulted from repeated exposure to PHMG-P persisted in mice 292 days after the last exposure, which is the equivalent of 26 years in humans.

The RNA-seq data showed that the mice in the PHMG-P-treated group showed an elevated expression of genes related to inflammatory response, regulation of cytokine production, leukocyte chemotaxis and migration, T cell proliferation and defense responses to other organisms. Proinflammatory cytokines (TNF, IL-1a, IL-6, IL-12b) and various chemokines (Ccl2, Ccl3, Ccl7, Ccl8, Ccl9, Ccl12, Cxcl2, Cxcl3, Cxcl5) were highly differentially expressed in the lung tissue of mice following instillation of PHMG-P. The inflammatory response was not resolved even 292 days after the last PHMG-P exposure. Persistent inflammation of the respiratory tract is a well-known pathogenic and toxic mechanism of PHMG-P [41]. Eventually, persistent inflammation in the lungs leads to pulmonary fibrosis through the extracellular matrix (ECM) deposition of fibrotic tissue [42]. The results support the upregulation of variable inflammation-related pathway including the migration of leukocytes and monocytes. 

Macrophages are actively involved in inflammatory and repair responses. Macrophages can be categorized into several subsets with distinct functions, including lung resident macrophages and recruited macrophages, M1 and M2 macrophages. Resident alveolar macrophages populate the lung during embryogenesis and are capable of self-renewal [43,44], while recruited macrophages originate from the bone marrow and move to the site of inflammation and undergo apoptosis [36,45]. Mould et al. (2019) [36] reported that recruited macrophages were the most distinct population and had the highest expression of M1 genes at the peak of inflammation, whereas resident macrophages expressed the highest levels of M2 genes during homeostasis and resolving inflammation. M1 macrophages produce proinflammatory mediators and eliminate bacteria or viruses from the host. M2 macrophages have an anti-inflammatory function and are involved in wound healing and repair [46]. We evaluated the expression of marker genes for each subset of macrophages in the PHMG-P-treated group to characterize the function of the macrophage subsets. Both resident and recruited macrophage marker genes were upregulated in the PHMG-P-treated group compared to the control group. Ccl2 plays an important role in recruiting monocytes in inflamed sites through the CCL2/CCR2 axis [47]. In this study, Ccl2 mRNA was markedly increased in the PHMG-P-treated group (3.26-fold change and *p* < 0.01), indicating that inflammation was not yet resolved. These results indicate that the infiltration of recruited macrophages was persistent, but that the repair process was more dominant in the PHMG-P-treated group. However, this macrophage sub-grouping has some limitations. First, RNA was isolated from the lung tissue, not macrophage population. Second, the markers of resident macrophages/recruited macrophages or M1/M2 macrophages are expressed by other cells in the lung like the dendritic cells, epithelial cells, or other immune cells. Another experiment involving, for example, flow cytometry analysis using bronchoalveolar lavage cells, is needed to validate these results. Nevertheless, these results may provide clues to support the notion that macrophages play an important role in PHMG-P induced pathogenesis. 

Next, an increase in neutrophil marker genes and neutrophil infiltration were detected in the PHMG-P-treated group. Neutrophils play an important role in acute inflammation and are usually depleted through phagocytosis by macrophages. Therefore, the influx and persistence of neutrophils in the lungs of mice with long-term recovery after last dose was significant. Our RNA-seq data showed that neutrophil attractants, namely, *Cxcl2* (2.56-fold change and *p* < 0.01) and *Cxcl5* (11.07 fold change and *p* < 0.01), were highly expressed in the PHMG-P-treated group. Elevated levels of *Itgam*, *Itgb2*, *Ltf* and *CD177* mRNA were associated with an increased number of neutrophils [48]. In the histopathological examination, neutrophils and macrophages were observed around cholesterol crystal in PHMG-P-treated group (Figure 1g). Cholesterol crystals are one of the danger signals released from damaged and dying cells and cause the recruitment of neutrophils and macrophages. These immune cells phagocytose the crystals and evoke an inflammatory response by activating toll-like receptors (TLRs) or NLRP3 [49,50]. The phagocytosed cholesterol crystals destabilize the lysosomal membranes and induce the leakage of lysosomal protease cathepsin B into the cytoplasm, leading to the activation of NLRP3 inflammasome and secretion of interleukin (IL)-1, IL-18 and IL-33 [51,52]. In this study, phagosome- and lysosome-associated genes such as *Acp5*, *Atp6v0d2*, *Ctsb*, *Ctsk* and *Rab7b* were significantly upregulated in the PHMG-P-treated group. These results indicate that persistent inflammation caused by the retention of PHMG-P and subsequent sterile inflammation might lead to the recruitment of various immune cells and the delayed resolution of inflammation even after long-term recovery. 

Chronic inflammation results in the apoptosis of parenchymal cells, the release of fibrotic cytokines and proteases such as MMPs, TIMPs and growth factors and leads to tissue destruction and fibrosis. EnrichR analysis showed the elevated expression of fibrosis-associated genes including *IL-12b*, *Hmox1*, *Ccl3*, *IL-6*, *Cxcl2*, *TNF*, *Ccl4*, *Plau*, *Timp1*, *Spp1*, *Ccl2* and *Muc5b*. Urokinase plasminogen activator (uPA, gene name: *Plau*) is a serine protease which converts plasminogen to plasmin. Plasmin contributes to tissue remodeling by degrading the ECM or activating MMPs [53]. Schuliga et al. reported that uPA was highly expressed in lung fibroblasts and epithelial cells in patients with idiopathic pulmonary fibrosis (IPF) and uPA-derived plasmin perpetuated fibrosis by stimulating the proliferation of lung fibroblasts and increased IL-6 production [54]. In addition, arginase (Arg)1 (3.85-fold change and *p* < 0.01) and Arg2 (1.79-fold change and *p* < 0.01), which are known to be involved in the development of lung fibrosis, were increased in the PHMG-P-treated group. Arginase hydrolyzes arginine to urea and ornithine. Ornithine is used for the synthesis of proline which is a component of collagen. Endo et al. (2003) [55] reported that the enhanced expression of Arg1 and Arg2 mRNA and proteins were observed from 5 days to 10 days after bleomycin treatment when collagen 1 mRNA and hydroxyproline were upregulated. In addition, Arg1 competes with nitric oxide (NO) synthase for arginine and suppresses NO production, which plays an important role in killing pathogens, cytotoxic actions and bronchodilation by inducing airway smooth muscle relaxation [56,57].

Interestingly, cellular senescence-associated genes including *Ccna1* (2.54-fold change and *p* < 0.05), *Ccnd1* (2.12-fold change and *p* < 0.01), *Cdkn1a* (2.27-fold change and *p* < 0.01), *Cdkn2a* (5.96-fold change and *p* < 0.01, *Gadd45g* (2.68-fold change and *p* < 0.01) and serpine1 (3.80-fold change and *p* < 0.01) were significantly induced. Cellular senescence is a permanent cell cycle arrest, which leads to the loss of regenerative capacities in response to cellular stress [58]. Induction of cellular senescence in fibrosis was shown by various groups such as Aoshiba et al. (2003) [59], Schafer et al. (2017) [60] and Maremanda et al. (2020) [61]. Persistent infiltration of immune cells exacerbates lung damage and subsequent repair demands may not be fulfilled due to loss of regenerative capacities of senescent cells, leading to abnormal lung structure and function.

Next, genes associated with lung structure and function were markedly reduced in the PHMG-P-treated group and this has not been reported yet. The PHMG-P-treated group showed downregulated gene expression associated with epithelial and endothelial cell development, regulation of epithelial and endothelial cells differentiation, endothelial cell migration and angiogenesis (Figure 3a). The molecular mechanisms of vascular remodeling in pulmonary fibrosis have been studied and alterations of micro-vessels were apparent [62,63]. However, some controversies regarding the relationship between lung fibrosis and angiogenesis exist. Previous studies have proposed that an increase in angiogenesis was observed in fibrotic lungs. However, recent studies support that angiogenesis may be decreased [62,63,64]. Murray et al. showed that decreased angiogenic mediators such as VEGF is observed in IPF patients. In addition, IPF patients with high levels of circulating VEGF had good prognosis and VEGF overexpressing mice showed diminished bleomycin-induced mortality and fibrosis through cytoprotective effects on epithelial cells [63]. In this study, numerous genes involved in endothelial cells development, differentiation and angiogenesis such as *Vegfa*, *Eng*, *Notch 4*, *Angpt1*, *Ednrb* and *Wnt7b*, were significantly decreased in the PHMG-P-treated group. 

The expression of genes associated with muscle structure development, muscle tissue development and smooth muscle contraction (*Actc1*, *Adra1a*, *Cav1*, *Cav2*, *Gucy1a1*, *Atp1a2*, *Npnt* and *Dock4*) was also significantly downregulated. Lungs contain smooth muscle cells [65] in bronchi and blood vessels [66]. Smooth muscle cells surround the lumen of the bronchi. This airway smooth muscle is indispensable for respiration because it protects the airway structure and assists exhalation by preventing airway collapsing or distortion from obstruction by external compression and mucus propulsion using peristaltic contraction [67]. Airway smooth muscles switch reversibly between a contractile phenotype and a more immature, proliferative phenotype [68]. Mature and contractile airway smooth muscle cells have a large number of caveolae and associated proteins, namely, caveolin-1 (Cav1) and caveolin-2. Caveolae are localized in the close proximity to the sarcoplasmic reticulum, mitochondria, or G-protein coupled receptors, which are important in intracellular Ca^2+^ influx, facilitating the contractile function of airway smooth muscles. Decreased Cav1 by small interfering RNA induce airway smooth muscle proliferation and increased airway smooth muscle mass augment thickening of the airway wall [69]. In addition, Cav1 has proapoptotic and antifibrotic features. Wang et al. (2006) [70] and Jin et al. (2011) [71] revealed that Cav1 was reduced in patients with IPF and Cav1 gene transfer ameliorates the fibrotic process by modulating TGF-β1-mediated Smad signaling and suppressing ECM production. Diminished Cav1 was also observed in patients with chronic obstructive pulmonary disease (COPD), asthma and cystic fibrosis [69]. 

The TGF-β pathway was shown to be a major pathway in fibrotic remodeling programs as a shared fibrotic signaling response among various fibrosis mechanisms [72]. Based on our RNA-seq results, PHMG-P seemed to use the TGF-β pathway as the interconnected pathway between persistent inflammation and the downregulation of the muscle and vascular systems.

The NO-soluble guanylate cyclase (sGC)-cyclic guanosine monophosphate (cGMP) pathway is involved in airway smooth muscle relaxation. Guanylate cyclase 1 soluble subunit alpha 1 (Gucy1a1) was significantly decreased in PHMG-P-treated group, which might lead to elevated smooth muscle tone and airflow limitation in the lungs [73,74]. The aforementioned decrease in NO synthase expression and NO production, which resulted from increased Arg1 expression, also participated in reduced airway smooth muscle relaxation. In addition, the sGC-cGMP pathway plays an antifibrotic role. Elevated cGMP inhibited pulmonary and renal fibrosis via TGF-β1 suppression and the inhibition of fibroblast-to-myofibroblast differentiation [75]. Hall et al. (2019) [76]. reported that sGC agonist showed anti-inflammatory and antifibrotic effects by suppressing the transformation of stellate cells into myofibroblasts in models of nonalcoholic steatohepatitis. The results of the present study showed that exposure to PHMG-P decreased angiogenesis-, muscle structure- and smooth muscle contraction-associated genes, which contributed to the interruption of airway remodeling and lung dysfunction [77]. 

Cytokeratin 14 (Krt14) was one of the highly upregulated genes in the PHMG-P-treated group. Cytokeratins are cytoplasmic intermediated filament proteins abundant in epithelial cells and different cytokeratins are expressed depending upon the cell type [78]. Lung basal cells, relatively undifferentiated epithelial cells, can regenerate and repair lung cells in response to injury and these cells express *p63, Krt5* and *Krt14* [79,80]. Unlike p63^+^Krt5^+^, p63^+^Krt14^+^ is rarely found in the distal airways, especially alveolar epithelium, in healthy lungs. In addition, p63^+^Krt5^+^cells co-express alveolar or bronchiolar differentiation markers (aquaporin 5 for alveolar type I cells or pro-surfactant protein C for alveolar type II cells), which indicate that these cells have the capacity to differentiate into proper epithelial cell types, whereas p63^+^Krt14^+^ cells did not co-express these differentiation markers [80]. Krt14 is highly upregulated in proliferating cells including hyperplastic alveolar cells seen in the diffuse alveolar damage of acute respiratory distress syndrome patients, bronchiolar metaplastic cells and in areas of more severe fibrosis in IPF patients [80,81]. Dakir et al. (2008) [82] reported that the constitutive expression of Krt14 induced multifocal airway cell hyperplasia, squamous metaplasia and carcinoma with the increasing age of Krt14 transgenic mice. In this study, hyperplastic alveolar epithelial cells were identified as prominent pathological feature and the gene expression of p63 (3.04-fold change and *p* < 0.01) and Krt14 (24.72-fold change and *p* < 0.001) was significantly upregulated in the PHMG-P-treated group. Taken together, a marked increase in Krt14 expression contributed to the proliferation or hyperplasticity of airway cells. In addition, these hyperplastic epithelial cells, especially type II epithelial cells, seem to lead to unfavorable outcomes. They produced and secreted excessive surfactant, leading to the formation of crystal clefts and the accumulation of a proteinaceous substance in the alveoli, which contributed to persistent inflammation, airway obstructions, abnormal gas exchange, dyspnea and reduced lung function. 

Mice with long-term recovery after PHMG-P treatment showed leukocyte chemotaxis and migration, fibrosis and bronchioloalveolar hyperplasia. The gene expression profiling results were consistent with these pathological findings and predicted aberrant structural changes and lung dysfunction, thus assisting in elucidation of the pathogenesis of PHMG-P. When PHMG-P reaches the alveoli, it is phagocytized by infiltrated neutrophils and macrophages. Immune cells produce a variety of cytokines and chemokines and reactive oxygen species to remove PHMG-P from the body. However, PHMG-P is not readily removed and irritates epithelial and immune cells. Whether PHMG-P is cleared from the body or not, sterile inflammation follows. Danger signals evoke an inflammatory response, leading to persistent inflammation and dysregulated repair, culminating in fibrosis. In addition, PHMG-P interrupts the development of the muscle and vascular systems, leading to delayed tissue repair and lung dysfunction. The translatability of this work in humans must be checked in a variety of ways, for example, by future epidemiological studies on the long-term effects and potential adverse effects of PHMG-P in humidifier disinfectant-associated disorders patients’ later in life. This study was achieved using male mice only. Further studies are needed to verify if similar results will be replicated in female mice.

## Figures and Tables

**Table 1:** Quantitative histopathologic evaluation of lung tissues in the PHMG-P-treated and the control groups.

Histopathological Findings	Control	PHMG-P
Foamy macrophage aggregation	0.00 ± 0.00	1.25 ± 0.96
Brochiolo–alveolar hyperplasia	0.00 ± 0.00	2.50 ± 0.58
Interstitial fibrosis	0.00 ± 0.00	1.75 ± 0.96
Alveolar/foamy/pigmented, diffuse macrophage	0.00 ± 0.00	2.25 ± 1.26
Cholesterol cleft	0.00 ± 0.00	2.00 ± 0.00
Alveolar proteinaceous substance	0.00 ± 0.00	1.75 ± 1.26
Lymphocytes infiltration	0.00 ± 0.00	1.75 ± 0.50

**Table 2:** Top 10 genes significantly upregulated by PHMG-P treatment.

Genes	Description	Fold Change	Volume	*p*-Value
*Ltf*	Lactotransferrin	275.95	5.57	1 × 10^−4^
*Gp2*	Glycoprotein 2 (zymogen granule membrane)	32.01	1.99	5 × 10^−4^
*Krt14*	Keratin 14	24.72	0.75	2 × 10^−3^
*Gpnmb*	Glycoprotein (transmembrane) nmb	24.70	4.33	4 × 10^−5^
*Mmp12*	Matrix metallopeptidase 12	20.84	3.23	1 × 10^−4^
*Bpifa1*	BPI fold containing family A, member 1	16.73	9.25	2 × 10^−3^
*Saa3*	Serum amyloid A 3	16.33	2.88	5 × 10^−4^
*Retnla*	Resistin-like alpha	16.30	7.13	1 × 10^−4^
*Jchain*	Immunoglobulin joining chain	14.74	6.88	8 × 10^−3^
*Cd177*	CD177 antigen	14.30	3.75	5 × 10^−3^

**Table 3:** Top 10 genes significantly downregulated by PHMG-P treatment.

Genes	Description	Fold Change	Volume	*p*-Value
*Car3*	Carbonic anhydrase 3	−26.88	1.42	1 × 10^−2^
*Cfd*	Complement factor D (adipsin)	−22.98	1.76	1 × 10^−2^
*Bex4*	Nerve growth factor receptor-associated protein 3, Brain expressed X-linked 4	−9.38	2.11	5 × 10^−3^
*Bex2*	Brain expressed X-linked 2	−9.05	3.08	7 × 10^−5^
*Hist1h2br*	Histone cluster 1 H2br	−7.15	0.41	1 × 10^−2^
*Fabp1*	Fatty acid binding protein 1, liver	−6.24	3.16	3 × 10^−4^
*Igfbp3*	Insulin-like growth factor binding protein 3	−5.71	3.76	7 × 10^−3^
*Slc7a10*	Solute carrier family 7, member 10	−5.57	2.38	8 × 10^−4^
*Glp1r*	Glucagon-like peptide 1 receptor	−5.46	3.94	2 × 10^−3^
*Cidec*	Cell death-inducing DFFA-like effector c	−5.21	1.98	3 × 10^−3^

## Data Availability

Data is contained within the article or supplementary material.

## Supplementary Materials

The following are available online at https://www.mdpi.com/article/10.3390/toxics9100253/s1, Figure S1: Scheme of experimental design; Figure S2: Top 10 pathways significantly upregulated or downregulated by PHMG-P treatment; Table S1: The list of primers used; Table S2: The list of differentially expressed genes; Table S3: Pathway enrichment for PHMG-P-treated group versus control group; Table S4: Top 10 biological pathways by EnrichR analysis; Table S5: The details of network among pathways; Table S6: Gene expression patterns of marcophage related markers by PHMG-P treatment.
