# Supplementary material for: Transcriptomic Analysis of Polyhexamethyleneguanidine-Induced Lung Injury in Mice after a Long-Term Recovery
Source: Toxics. 2021 Oct 8;9(10):253. doi: 10.3390/toxics9100253 (PMC8540838; doi:10.3390/toxics9100253)
Supplement: Supplementary file 1 [file toxics-09-00253-s001.zip › toxics-1391431-sm-final.pdf]

toxics

Supplementary Materials: Transcriptomic Analysis of Polyhexamethyleneguanidine-induced Lung Injury in Mice after a Long-term Recovery

Jeongah Song, Kyung Jin Jung, Jae-Woo Cho, Tamina Park, Su-Cheol Han and Daeui Park

Table S1. The list of primers used.

Gene name	Accession number	Forward primer	Reverse primer	Product size
Serpine1	NM_022415	CTTTGTCATCTCAGCCCGCA	TGAAGAGGATTGTCTCTGTCGG	98
Ccl2	NM_011333	AGGTGTCCCAAAGAAGCTGTA	ATGTCTGGACCCATTCCTTCT	85
Mmp12	NM_001320076	CACAACAGTGGGAGAGAAAA	AGCTTGAATACCAGATGGGATG	130
Ptges	NM_022415	TCCTCGGCTTCGTGTACTCA	ATCCAGGCGATCAGAGGGTT	52
Arg1	NM_007482	CTTTCTCAAAAGGACAGCCTCG	GACCAGCTTTCCTCAGTGCT	66
Arg2	NM_022415	CTCCACGGGCAAATTCCTTG	AGAGAAAGGGGCTCCGACTA	72
Ctsb	NM_007798	GGCTCTTGTTGGGCATTTGG	ACTCGGCCATTGGTGTGAAT	71
Rab7b	NM_001311096	TAGGAAGTACCAGGGCACCG	GCCTGGAGAGAGCTTGATGG	61
Cav1	NM_001243064	GACCCCAAGCATCTCAACGA	AGATGCCGTCGAAACTGTGT	91
Gucy1a3	NM_021896	TTCCCGAAGAGAACCACAGC	ACAGTCACTTCGGAAGCAGG	117
Krt14	NM_001313956	CCGACCTGGAGATGCAGATT	GCCACCTCCTCGTGGTTC	73

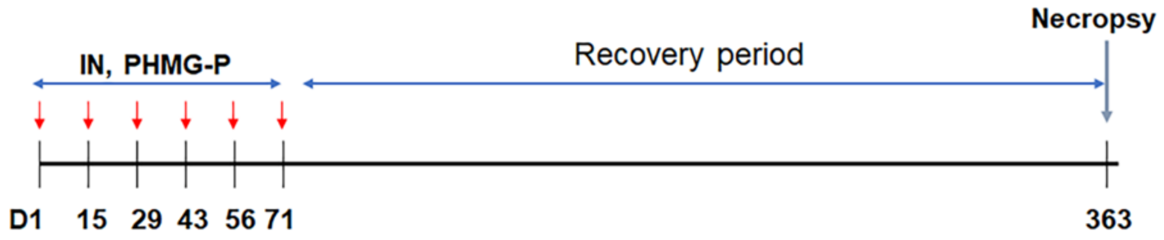

Figure S1. Scheme of experimental design. Mice were intranasally instilled with 0.3 mg/kg PHMG-P six times at 2-week intervals. The control group was treated with saline through the same route.

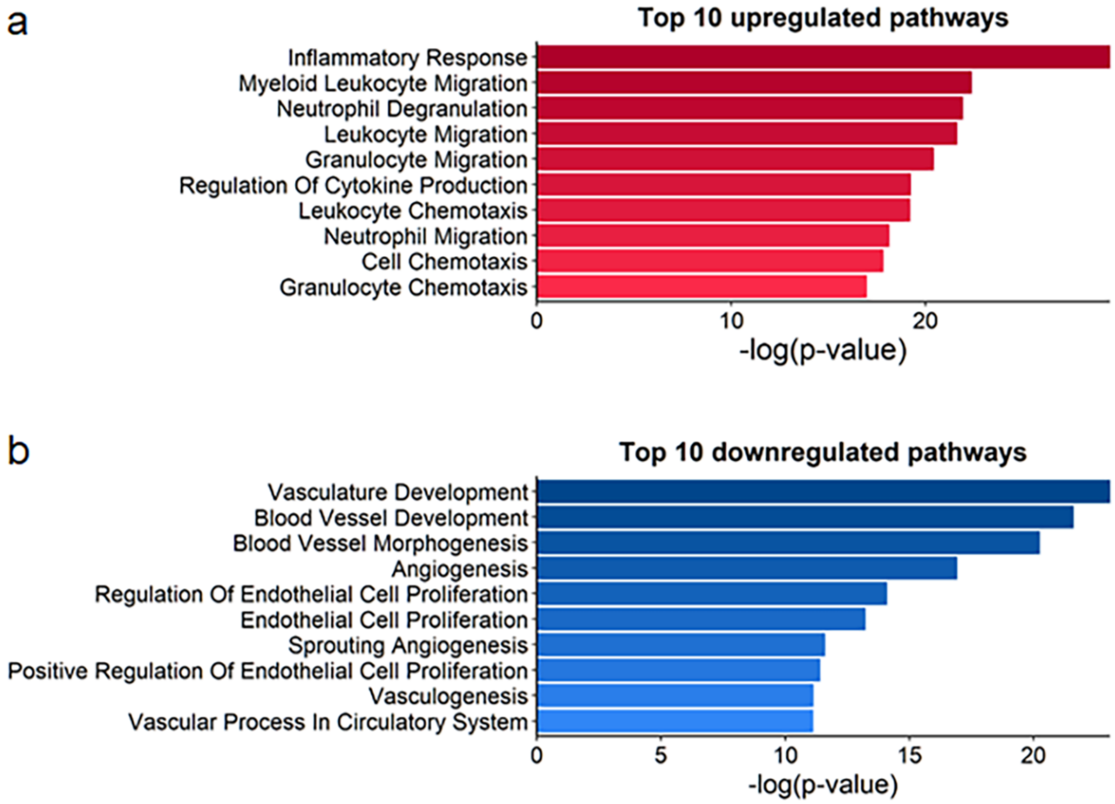

Figure S2. Top 10 biological pathways significantly changed by PHMG-P in EnrichR analysis.
